# EGFR Kinase Promotes Acquisition of Stem Cell-Like Properties: A Potential Therapeutic Target in Head and Neck Squamous Cell Carcinoma Stem Cells

**DOI:** 10.1371/journal.pone.0032459

**Published:** 2012-02-27

**Authors:** Eric L. Abhold, Alan Kiang, Elham Rahimy, Selena Z. Kuo, Jessica Wang-Rodriguez, Jay Patrick Lopez, Katherine J. Blair, Michael Andrew Yu, Martin Haas, Kevin T. Brumund, Xabier Altuna, Andrew Patel, Robert A. Weisman, Weg M. Ongkeko

**Affiliations:** 1 Division of Otolaryngology-Head and Neck Surgery, Department of Surgery, University of California San Diego, San Diego, California, United States of America; 2 Veterans Administration Medical Center and Department of Pathology, University of California San Diego, San Diego, California, United States of America; 3 Moores Cancer Center, University of California San Diego, San Diego, California, United States of America; 4 Hospital Universitario Donostia, San Sebastian, Spain; Penn State Hershey Cancer Institute, United States of America

## Abstract

Members of the EGFR/ErbB family of tyrosine kinases are found to be highly expressed and deregulated in many cancers, including head and neck squamous cell carcinoma (HNSCC). The ErbB family, including EGFR, has been demonstrated to play key roles in metastasis, tumorigenesis, cell proliferation, and drug resistance. Recently, these characteristics have been linked to a small subpopulation of cells classified as cancer stem cells (CSCs) which are believed to be responsible for tumor initiation and maintenance. In this study, we investigated the possible role of EGFR as a regulator of “stemness” in HNSCC cells. Activation of EGFR by the addition of EGF ligand or ectopic expression of EGFR in two established HNSCC cell lines (UMSCC-22B and HN-1) resulted in the induction of CD44, BMI-1, Oct-4, NANOG, CXCR4, and SDF-1. Activation of EGFR also resulted in increased tumorsphere formation, a characteristic ability of cancer stem cells. Conversely, treatment with the EGFR kinase inhibitor, Gefinitib (Iressa), resulted in decreased expression of the aforementioned genes, and loss of tumorsphere-forming ability. Similar trends were observed in a 99.9% CD44 positive stem cell culture derived from a fresh HNSCC tumor, confirming our findings for the cell lines. Additionally, we found that these putative cancer stem cells, when treated with Gefitinib, possessed a lower capacity to invade and became more sensitive to cisplatin-induced death *in vitro*. These results suggest that EGFR plays critical roles in the survival, maintenance, and function of cancer stem cells. Drugs that target EGFR, perhaps administered in combination with conventional chemotherapy, might be an effective treatment for HNSCC.

## Introduction

Head and neck squamous cell carcinoma (HNSCC) is the 6^th^ leading cancer in the world and in many developing countries is the second most common cancer. In the United States, 30,000–40,000 cases of head and neck cancer are diagnosed annually. Despite advances in the field of cancer in general, survival rates have not improved significantly in more than 30 years [1]. Although the molecular biology of HNSCC is poorly understood, it is clear that deregulation of several kinases is implicated in the pathogenesis of this disease. Most prominent among these kinases is epidermal growth factor receptor (EGFR), a receptor tyrosine kinase which is overexpressed in a variety of solid tumors, including the majority of HNSCC, and whose expression is correlated with a poor clinical outc`ome [2], [3]. Signaling through EGFR has been implicated in various processes that contribute to cancer initiation and progression, including cell proliferation, drug resistance, tumorigenicity, invasion, and metastasis [4], [5], [6], [7]. These same properties have been attributed to cancer stem cells (CSC), a rare subpopulation of self-renewing cancer cells with the ability to proliferate extensively and recapitulate tumors [8].

Cancer stem cells have been identified in leukemia as well as in several solid tumors. In HNSCC, Prince et al demonstrated that the cell surface marker CD44 could be used to sort and isolate putative cancer stem cells [9]. These CD44+ lineage negative (Lin-) cells had a primitive cellular morphology and co-stained with the basal cell marker Cytokeratin5/14, whereas the CD44− cells resembled squamous epithelium and expressed the differentiation marker involucrin. They showed that tumors arising from CD44+ cells reproduced the original tumor heterogeneity and could be serially passaged, suggesting that CD44+ cells were stem cells, as they had the properties of differentiation and self-renewal.

Cancer stem cells are thought to arise from progenitor cells that have acquired stem cell properties through aberrant behavior of key regulatory genes, proto-oncogenes and tumor suppressors. Proto-oncogenes, including those that play a key role in HNSCC tumorigenesis such as EGFR and Akt, are thought to promote regenerative capacity by promoting stem cell function. Conversely, tumor suppressors inhibit regenerative capacity by promoting cell death or senescence in stem cells, but also protect against cancer [10]. Members of the EGFR/ErbB family of receptor tyrosine kinases are proto-oncogenes and are highly expressed in many cancers of epithelial origin including HNSCC. EGFR has several known growth factor ligands including epidermal growth factor (EGF), and TGFα, which upon binding to the extracellular domain of EGFR causes the activation of a number of downstream effectors involved in the ras/raf-1/mitogen-activated protein kinase, the phosphatidylinositol-3-kinase, and the phospholipase C pathways [11], [12]. Activation of these genes results in the expression of other proteins responsible for cell growth coordination. Overexpression of EGFR is observed in 42% to 80% of HNSCCs studied [13]. EGFRvIII, a constitutively active mutant form of EGFR, is common in HNSCC as well as in other cancers known to be highly aggressive. Grandis et al demonstrated that EGFR overexpression provided independent prognostic value for both local control and survival in 91 head and neck squamous carcinoma patients treated with surgical resection +/− postoperative radiotherapy [14]. EGFR overexpression was also associated with both an increased risk of local relapse and an adverse overall survival, independent of tumor stage.

Due to the link between EGFR and poor prognosis, we speculated that EGFR may also regulate stem cell characteristics in HNSCC, chiefly the ability to maintain self-renewal despite constant proliferation. If this hypothesis were correct, then constitutive activation of EGFR in normal stem cells or progenitor cells could ostensibly lead to cancer. A recent clinical study demonstrated a link between EGFR and ErbB2 inhibition and reduced CD44^+^/CD24^low^ expression, the cancer stem cell marker in breast cancer [15], [16]. This observation has subsequently been partially confirmed at the basic science level as ErbB2 has been demonstrated to regulate the mammary/stem progenitor cell population [17]. Here, we hypothesized that aberrant activation of EGFR is one of the key steps that has resulted in the pathogenesis of HNSCC cancer stem cells. In its behavior as an oncogene, EGFR can promote acquisition of cancer stem cell-like properties in HNSCC cancer cells and may determine the fate of progenitor cells, thus making it an attractive therapeutic target.

## Materials and Methods

### Ethics statement

Cultures used in this study (JLO-1) were derived in accordance with the ethics board of Hospital Donostia, San Sebastian, Spain. Per the hospital's ethics board, consents are waived when tissue is obtained anonymously and de-identified. All data analysis associated with this culture was performed anonymously. Hospital Donostia, San Sebastian approved this procurement of tissue including the waiver of consent.

### Cell lines and Cell Culture

The HNSCC cell lines used were UMSCC22B, a gift from Dr. Tom Carey, University of Michigan and HN-1, a gift from Dr. J.S. Gutkind, National Institute for Dental and Craniofacial Research. HN-1 was derived from a tongue squamous carcinoma [18], while 22B was derived from a metastatic cervical lymph node of a patient with hypopharygeal cancer [19]. The cells were cultured in DMEM supplemented with 10% fetal calf serum, 2% penicillin/streptomycin, and 2% L-glutamate (GIBCO) and maintained at 37°C in a humidified 5% CO_2_/95% air atmosphere. JLO-1 is a putative cancer stem cell culture derived from a fresh laryngeal tumor, which was obtained from an anonymous patient undergoing surgical resection at Hospital Donostia (San Sebastian, Spain) in accordance with their ethics board. Following isolation by both flow cytometry (CD44+) and selective colony formation on laminin-coated plates, putative cancer stem cell cultures were maintained in keratinocyte serum-free media (Invitrogen, Carlsbad, CA) supplemented with growth factors (EGF and FGF), L-glutamine (10 mg/mL, Invitrogen, Carlsbad, CA), and gentamycin (Invitrogen, Carlsbad, CA). Cells were grown at 37°C at 5% O_2_ and 10% CO_2_. EGF ligand (R&D Systems) and the EGFR-specific tyrosine kinase inhibitor Gefitinib (ZD1839) were dissolved in DMSO at 10 mMol/L, stored at −20C,and diluted with DMEM before use.

### Quantitative RT-PCR

Cells were harvested two days after being passaged at about 70–80% confluence. Total cell lysate was collected and RNA was extracted using an RNeasy kit (Qiagen). cDNA was synthesized using Superscript III Reverse Transcriptase (Invitrogen, Carlsbad, CA) as per the manufacturer's instructions. Real-time PCR reaction mixes were prepared using Power SYBR Green (Applied Biosystems, Foster City, CA), and run on the 7300 Real-time PCR System (Applied Biosystems) using the following program: 95°C for 10 min, 95°C for 30 s, and 60°C for 1 min, for 40 cycles. Results were analyzed using the ΔΔCt method. Experiments were done in technical triplicates and were repeated at least twice independently. GAPDH gene expression was measured as endogenous control. Primers were custom ordered (Eurofins MWG Operon, Huntsville, AL) using the following sequences: SDF-1 (FOR CTGCCTCAGCGACGGGAAGC – REV TCGAGTGGGTCTAGCGGAAAGT), CXCR-4 (FOR TGTGACCGCTTCTACCCCAATGAC – REV GGACAGGATGACAATACCAGGCAG), and beta-actin (FOR CGCTGGATTTTCAAAACAGT – REV CTGAGGAGCAGCTTCAGTCC). Oct-3/4 forward 5′- GCAAAGCAGAAACCCTCGTGC-3′ reverse 5′- ACCACACTCGGACCACATCCT-3′. Nanog forward 5′- GATTTGTGGGCCTGAAGAAA-3′ reverse 5′- TTGGGACTGGTGGAAGAATC-3′. CD44 forward 5′- ACACCACGGGCTTTTGACCAC-3′ reverse 5′- AGGAGTTGCCTGGATTGTGCTTG-3′. BMI-1 forward 5′- TCCACAAAGCACACACATCA-3′ reverse 5′- CTTTCATTGTCTTTTCCGCC-3′.

### Western Blotting

Cell lines were lysed in detergent containing 1% NP40,150 mmol/L NaCl, 1 mmol/L EDTA, 0.1 mmol/L phenylmethylsulfonyl fluoride,1 Ag/mL leupeptin,and 1 Ag/mL aprotinin, and protein levels were determined using the Bradford protein assay method (Bio-Rad Laboratories). Thirty micrograms of total protein was separated on 10% SDS-PAGE gels and transferred to nitrocellulose membranes. Membranes were blocked with 5% BSA in TBS with Tween-20 solution for 1 hour at room temperature and incubated with primary antibodies (1∶1000) in 5% BSA in TBS-T overnight at 4°C. After several washes in TBST, membranes were incubated for 1 h at room temperature with the appropriate secondary antibody (1∶10,000). The membranes were again washed several times and the protein-antibody complexes were detected using Luminol Reagent (Thermo Scientific). Antibodies used for blotting included anti-BMI (ABCam), anti-CD44 (ABCam), Anti-Oct4 (ABCam), Anti-Nanog (Cell Signaling), Anti-SDF1 (ABCam), Anti-CXCR4 (ABCam), Anti-EGFR (SantaCruz Biotechnology), and Anti-B-Actin (Cell Signaling).

### Sphere Formation Assay

Cells were seeded in a 24-well low adhesion plate (Corning Inc, Lowell, MA) at a density of 1000 cells/well and an initial volume of 500 ul as described by Dontu et al [20]. Spheres were grown in DMEM F12 medium (Gibco) supplemented with Vitamin B27, 20 ng/ml FGF, and 4 ug/ml of heparin. Samples were subsequently treated with 0, 10, 50, or 100 ng/ml of epidermal growth factor (R&D Biosystems) or 2 µM Gefitinib. EGF and FGF were replenished every three days. Aside from the addition of heparin, cells were agitated daily to minimize clumping. Spheres were counted and photographed after 10 days of continuous ligand or inhibitor exposure. Spheres were monitored under the microscope daily to ensure that they were derived from single cells and that they did not become confluent during the experiment.

### Immunofluorescence

To determine the effect of gefitinib on the expression of CD44 in our putative cancer stem cell culture, JLO-1, cells were incubated with 2 µM gefitinib for 24 hours. The cells were fixed with 4% paraformaldehyde and blocked in goat serum at room temperature prior to incubation with the monoclonal CD44 antibody (R&D Systems, Minneapolis, Minnesota). Cells tagged with the CD44 antibody were then incubated with goat anti-rabbit fluorescein isothiocyanate (FITC)^−^conjugated secondary antibody (Chemicon). Cells were counterstained with 4,6-diamidino-2-phenylindole (DAPI). Images were taken at the original magnification of 40×. Fluorescent images were obtained using a Leica inverted fluorescence microscope (model DMIRE2; Leica Microsystems, Deerfield, Illinois). The computer software Simple PCI (Compix Inc, Sewickley, Pennsylvania) was used for image capture.

### Invasion Assay

Invasion of JLO-1 cells was measured using a Matrigel invasion assay (Becton Dickinson, Bedford, MA). Transwell inserts of 8 µm pore size were coated with a final concentration of 1 mg/mL of Matrigel in cold serum-free DMEM. Cells were trypsinized, and 1×10^5^ cells were added in triplicate wells. The lower chamber of the transwell was filled with 750 µl of culture media containing 0.5% serum as a chemoattractant, along with the treatment of either 20 ng/ml EGF or 20 µM Gefitinib and allowed to incubate at 37°C for 24 hours. Invading cells on the lower surface that passed through the filter were fixed and stained using crystal violet in gluteraldehyde and photographed.

### Flow Cytometry Analysis

JLO-1 cells were trypsinized and incubated with either the monoclonal CD44 antibody conjugated to the fluorescent dye phycoerythrin (R & D Systems, Minneapolis, Minnesota) or the monoclonal EGFR antibody conjugated to the fluorescent dye allophycocyanin R & D Systems, Minneapolis, Minnesota). Samples were analyzed by flow cytometry and gated in comparison to an unlabeled control.

### MTS Assay

JLO-1 cells were plated into a 96 well flat-bottom tissue culture plate (Falcon) at a density of 5,000 cells per well. After a 24 hour plating period, half of the wells were treated with 2 µM Gefitinib and incubated for 48 hours. The control and Gefitinib-treated cells were subsequently exposed to one of several doses of cisplatin ranging from 0–20 µM. After a 24 hour incubation period, the cells were analyzed for chemoresistance using an MTS proliferation assay (Promega) in accordance with the manufacturer's protocol. All assays were performed in triplicate.

## Results

### EGFR activation regulates the expression of cancer stem cell markers in HNSCC cell lines

**Figure 1:**
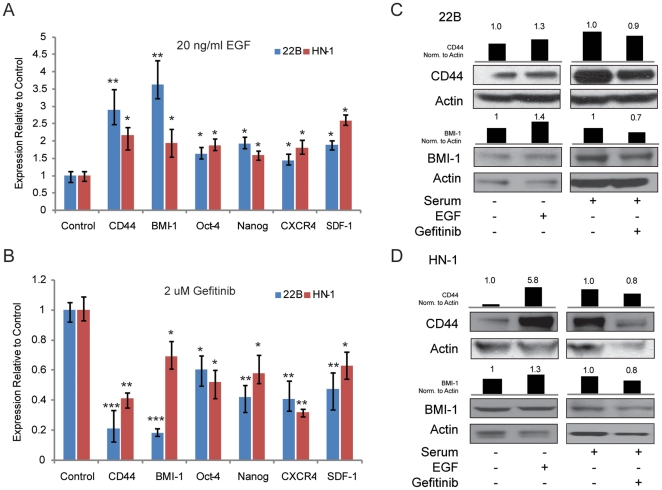
EGFR signaling regulates the expression of stem cell and metastatic markers in the HNSCC cell lines 22B and HN-1. (A) Treament with 20 ng/ml EGF induced the expression of CD44, BMI-1, Oct-4, Nanog, CXCR4 and SDF-1alpha. (B) Treatment with 2 µM of Gefitinib lowered the expression of CD44, BMI1, Oct-4, Nanog, CXCR4 and SDF-1alpha. Error bars represent SD. * indicates p<.05, *** indicates p<.01, *** indicates p<.001 (C) Effect of 20 ng/ml EGF or 2 µM Gefitinib on the expression of BMI-1 and CD44 protein in 22B cells. (D) Effect of 20 ng/ml EGF or 2 µM Gefitinib on the expression of BMI-1 and CD44 protein in HN-1 cells.

We first sought to determine whether EGFR might regulate the expression of a set of well-established cancer stem cell markers. CD44 and BMI-1 have been shown to indicate stem cell phenotype in HNSCC [9]. Oct-4 and Nanog are pluripotency and self-renewal markers in embryonic stem cells, but are also found to be overexpressed in many cancers. The CXCR4/SDF-1 axis is known to be responsible for invasion and metastasis in pancreatic cancer, and is a hematopoietic stem cell marker. 22B and HN-1 cells were serum starved overnight, treated with 20 ng/ml EGF ligand for 24 hours, and then harvested for total RNA or protein. Quantitative polymerase chain reaction was then used to compare expression levels between control and EGF treated cells (Figure 1a). EGF treated cells experienced 1.5 to 4-fold increases in expression of Bmi-1, CD44, Oct-4, Nanog, CXCR4 and SDF-1 mRNA. In contrast, cells treated with 2 µM Gefitinib, a small molecule inhibitor of EGFR, experienced 2 to 5 fold decreases in the same set of genes (Figure 1b). Using western blots, these results were verified at the protein level for the HNSCC stem cell markers BMI-1 and CD44 (Figure 1c, d). Relative levels of protein were quantified by performing densitometric analysis on each band and comparing the ratio of CD44 or BMI-1 to Actin between samples, with control samples set arbitrarily to 1. These results demonstrate that EGFR signaling might regulate cancer stem cell properties, as it induces the expression of genes responsible for self-renewal, pluripotency, and metastasis.

### Overexpression of EGFR induces the expression of cancer stem cell genes in HNSCC cell lines

**Figure 2:**
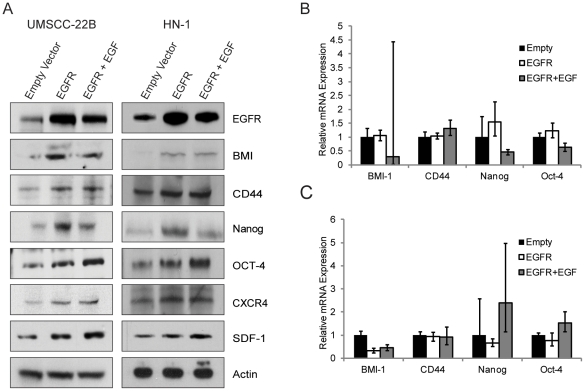
EGFR overexpression induces protein expression of cancer stem cell markers in HNSCC cell lines. (A) EGFR overexpression induces higher protein levels of stem cell and metastatic markers in the HNSCC cell lines 22B and HN-1. EGFR was transiently overexpressed in the cell lines 22B and HN-1, and protein levels were measured 48 hours later by western blotting. (B) No significant change in mRNA levels was observed in 22B cells upon EGFR overexpression, suggesting a potential post-transcriptional mechanism. (C) No significant change in mRNA levels was observed in HN-1 cells upon EGFR overexpression, suggesting a potential post-transcriptional mechanism.

EGFR is overexpressed in 90% of head and neck cancers [7]. To determine whether EGF is acting through EGFR or a secondary target, exogenous EGFR was overexpressed in both HNSCC cell lines in the presence of serum and exposed to either 0 or 20 ng/ml EGF ligand. EGFR overexpression alone resulted in a significant increase in all six CSC genes (Figure 2a). The addition of EGF ligand to EGFR-overexpressing cells had various effects on the six CSC genes, either increasing expression (CD44, Oct-4, and Sdf-1), having no appreciable effect (BMI1, CXCR4), or causing a decrease in expression (Nanog). These results suggest that BMI and CXCR4 may already have reached a maximum expression level. Furthermore, these results suggest that EGF ligand may also activate an opposing pathway controlling Nanog expression, leading to a slightly diminished increase in Nanog expression. Interestingly, no significant change in CD44, BMI-1, Nanog, and Oct-4 was observed at the mRNA level upon ectopic overexpression of EGFR in both UMSCC-22B and HN-1, suggesting involvement of a post-transcriptional mechanism (Figure 2b, c). Taken together, these results demonstrate that EGFR activity is responsible, via post-translational regulation, for the shown increase in the CSC gene expression.

### EGFR kinase regulates tumorsphere formation in HNSCC cell lines and putative HNSCC stem cells

**Figure 3:**
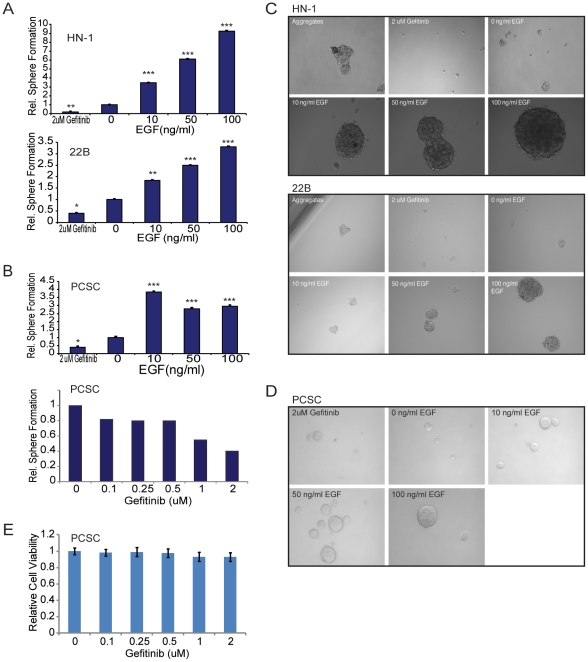
EGFR regulates tumorsphere formation in HNSCC cell lines and in a putative cancer stem cell culture. (A) EGF increased the rate of sphere formation in a dose-dependent fashion in 22B and HN-1 cells, while addition of 2 µM gefitinib significantly decreased sphere formation. (B) EGF increased the rate of sphere formation in JLO-1, a putative cancer stem cell culture. Error bars represent SEM. Gefitinib (0 to 2 µM) reduced sphere formation in a dose-dependent fashion in JLO-1. (C) Brightfield micrographs showing sphere formation in 22B and HN-1 after treatment with Gefitinib or EGF. (D) Brightfield micrographs showing sphere formation in JLO-1 after treatment with Gefitinib or EGF. (E) MTS cell viability assay demonstrating negligible effect of Gefitinib (Iressa) on cell viability at doses of up to 2 µM, showing that the observed decrease in sphere formation is not due to lower cell viability.

To further demonstrate the role of EGFR activity in regulating CSC properties, we determined whether EGFR could regulate tumorsphere formation. Spheres have been shown to be enriched for cancer stem cells, and are used as a measure of stemness in many tissues. A sphere formation assay was performed, as described in Dontu et al, for cells treated with either 0, 10, 50, or 100 ng/ml of EGF ligand or 2 µM Gefitinib (Figure 3a–d). 22B, HN-1, and a putative cancer stem cell culture (JLO-1) were seeded in a 24-well low adhesion plate at 1000 cells per well. The cells were treated for two weeks after which the spheres were counted and photographed. EGF ligand enhanced self-renewal capacity in both established HNSCC cell lines and our putative cancer stem cell line, as indicated by the dose dependent increase in sphere formation and sphere size. Meanwhile, Gefitinib exposure led to a significant decrease in sphere-formation ability. In order to ensure that the observed reduction in sphere formation was due primarily to inhibition of stem cell phenotype rather than decrease in cell viability, a MTS cell viability assay was performed to ensure that no significant reduction in cell viability occurred over a period of 7 days at the relevant doses of Gefitinib (Figure 3e).

### EGFR inhibition decreases CD44 expression in putative HNSCC stem cell culture

**Figure 4:**
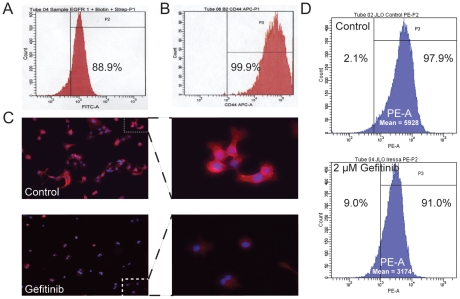
Gefitinib inhibits CD44 expression in putative cancer stem cells. (A) Flow cytometric analysis showing a high proportion (88.9%) of EGFR+ JLO-1 cells. (B) Flow cytometric analysis showing a high proportion (99.9%) of CD44+ JLO-1 cells. (C) Immunofluoresence microscopy showing the effect of 2 µM gefitinib on cell-surface CD44 expression in JLO-1. CD44 fluorescence (Red) is markedly reduced in the bottom two panels, suggesting that EGFR activity is essential for CD44 expression. (D) Flow cytometric analysis quantifying the change in CD44 expression and size of CD44-negative population upon treatment with Gefitinib.

We measured by flow cytometry the proportion of CD44 and EGFR-expressing JLO-1 cells. 88.9% showed high expression of EGFR (Figure 4a) and 99.9% had high expression of CD44 (Figure 4b). In order to further demonstrate the connection between EGFR activity and acquisition of stem cell properties, we looked at the effect of EGFR inhibition on expression of CD44 protein. Immunofluorescence analysis revealed that cell surface expression of CD44 was lowered dramatically in response to treatment with Gefitinib (Figure 4c). Flow cytometric analysis was also performed to verify the reduction in CD44 expression (Figure 4d). Flow analysis revealed a 0.5-fold reduction in mean PE fluorescence accompanied by an approximate 4.5-fold expansion (2.1% to 9.0%) of the CD44 negative population. Given that CD44 is the main stem cell marker for HNSCC, these results suggest that EGFR activation may be crucial for maintaining a cancer stem cell phenotype.

### EGFR inhibition sensitizes cells to cisplatin and blocks cellular invasion in a putative HNSCC stem cell culture

**Figure 5:**
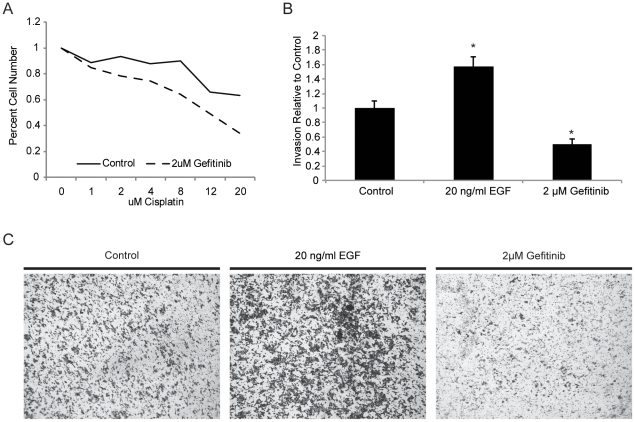
Gefitinib enhances cisplatin-induced death and lowers invasive capacity in putative cancer stem cells. (A) MTS assay comparing the survival curves of control JLO-1 to 2 µM gefitinib-treated JLO-1 when subjected to increasing doses of cisplatin. Gefitinib-treated JLO-1 lost their viability at noticeably lower doses in comparison to control cells. (B) Matrigel invasion assay comparing relative number of cells invaded between control, EGF-treated, and Gefitinib-treated JLO-1. (C) Representative photographs of the bottom surfaces of the invasion chambers, showing clear differences in the number of invaded cells between the three samples.

We performed an MTS assay to test the viability of Iressa-treated JLO-1 cells in the presence of cisplatin (Figure 5a). Our results showed that treatment with 2 µM of Iressa sensitized the cells to cisplatin. This suggests that Iressa may be effective in combination with conventional chemotherapy. In addition, matrigel invasion assays were performed to test the role of EGFR signaling in regulating cellular invasion (Figure 5b, c). Results showed that treatment with 20 ng/ml of EGF ligand increased invasion by 1.6-fold, while inhibition using 2 µM of Iressa lowered invasion by greater than 2-fold. These results are clinically relevant as EGFR expression has been positively correlated with poor clinical outcome. In addition, it shows that Gefitinib may be an effective agent for use in combination with conventional chemotherapy by promoting drug sensitization and inhibiting invasion.

## Discussion

Although it is known that EGFR facilitates cancer progression by inducing cell growth and proliferation, these effects alone may not be enough to overcome the natural barriers of senescence and terminal differentiation. It is likely that secondary mechanisms, in addition to mitotic stimulation, exist to circumvent such barriers and allow cancer cells to thrive indefinitely. Here we have shown that EGFR possibly fulfills that role, as EGFR activation promoted the acquisition and maintenance of various cancer stem cell characteristics including the expression of self-renewal markers and capacity to form tumorspheres. In addition, we have found that EGFR promotes invasion and chemoresistance in a putative cancer stem cell culture, suggesting that EGFR also regulates malignant behavior in cancer stem cells. These observations suggest that EGFR deregulation might be an important step in the conversion of adult stem cells or progenitor cells into malignant cancer stem cells.

One of the prevailing models is that cancer stem cells arise from progenitor cells that have obtained the ability to self-renew. Under normal development, EGFR is used to promote proliferation and self-renewal of adult stem cells, and becomes tightly regulated or inactivated as they differentiate into progenitor cells. In cancer, it is possible that mutations would cause EGFR to remain constitutively active even in differentiated cells, resulting in maintenance or acquisition of self-renewal properties. The most common mutant form, EGFRvIII, is a hallmark of highly aggressive cancers and is present in 42% of HNSCC tumors [21]. It was shown that HNSCC cell lines transfected with EGFRvIII show increased proliferation and decreased sensitivity to cisplatin-induced apoptosis. Furthermore, ectopically expressed EGFRvIII was found to inhibit differentiation and promote migration in both neural stem cells and neural progenitors [22], [23]. Our findings, taken together with these observations, suggest the possibility that aberrant activation of EGFR in cancer can lead to acquisition and maintenance of stem cell properties. Future work is needed to elucidate the downstream pathways that regulate EGFR-induced stemness, although it is likely that these effects are at least partly mediated by the PI3K/Akt pathway, which in turn regulates a variety of stem cell functions including proliferation, self renewal, invasion and drug resistance.

We observed that both activation and overexpression of EGFR resulted in increased expression of stem cell markers. Transient overexpression of EGFR in our cell lines increased the protein levels of stem cell markers CD44, Oct-4, Sdf-1, and Nanog. Addition of EGF simultaneously slightly lowered the expression of Nanog compared to overexpression of EGFR alone, indicating a possible negative feedback loop. A possibility is that the addition of EGF increases the expression of Oct-4 past a certain threshold, at which it begins to repress the Nanog promoter. This relationship has been well-demonstrated in embryonic stem cells [24]. Interestingly, EGFR overexpression resulted in increased expression of stem cell markers at the protein level but did not generate significant change in expression at the mRNA level, suggesting a post-transcriptional regulatory mechanism.

Recent studies suggest that specific targeting of cancer stem cells improves disease outcomes in mice [25], [26]. Our results suggest EGFR inhibition might be an effective method to target the cancer stem cell population in HNSCC. Thus, treatments that combine EGFR inhibitors with conventional chemotherapy will target both the bulk of the tumor and the cancer stem cell subpopulation, greatly reducing the possibility of relapse and secondary tumors. Additionally, our findings demonstrate that the role of EGFR in HNSCC extends beyond its traditional function as a promoter of cell growth, as it could regulate the key properties of cancer stem cells that are essential for cancer initiation and progression.
